# A Conserved Mechanism for Control of Human and Mouse Embryonic Stem Cell Pluripotency and Differentiation by Shp2 Tyrosine Phosphatase

**DOI:** 10.1371/journal.pone.0004914

**Published:** 2009-03-17

**Authors:** Dongmei Wu, Yuhong Pang, Yuehai Ke, Jianxiu Yu, Zhao He, Lutz Tautz, Tomas Mustelin, Sheng Ding, Ziwei Huang, Gen-Sheng Feng

**Affiliations:** 1 Programs in Signal Transduction, and Stem Cells and Regeneration, Burnham Institute for Medical Research, La Jolla, California, United States of America; 2 Department of Chemistry, Scripps Research Institute, La Jolla, California, United States of American; Istituto Dermopatico dell'Immacolata, Italy

## Abstract

Recent studies have suggested distinctive biological properties and signaling mechanisms between human and mouse embryonic stem cells (hESCs and mESCs). Herein we report that Shp2, a protein tyrosine phosphatase with two SH2 domains, has a conserved role in orchestration of intracellular signaling cascades resulting in initiation of differentiation in both hESCs and mESCs. Homozygous deletion of *Shp2* in mESCs inhibited differentiation into all three germ layers, and siRNA-mediated knockdown of *Shp2* expression in hESCs led to a similar phenotype of impaired differentiation. A small molecule inhibitor of Shp2 enzyme suppressed both hESC and mESC differentiation capacity. Shp2 modulates Erk, Stat3 and Smad pathways in ES cells and, in particular, Shp2 regulates BMP4-Smad pathway bi-directionally in mESCs and hESCs. These results reveal a common signaling mechanism shared by human and mouse ESCs via Shp2 modulation of overlapping and divergent pathways.

## Introduction

The establishment of human embryonic stem cell (hESC) lines has generated a great deal of excitement in the fields of stem cell biology and regenerative medicine [1]. It has been hoped that hESCs and somatic stem cells could be potential sources of cells to regenerate or rejuvenate damaged tissues. However the intracellular signaling mechanisms that regulate hESC pluripotency and differentiation are poorly understood, partly due to difficulties in manipulating this type of stem cells in vitro. In the past few years, much attention has been paid to transcription factors, such as Oct4, Sox2 and Nanog, which are essential for the establishment of human and mouse ES cell identity. Notably, these factors are found to co-occupy a substantial portion of their target genes [2], [3]. It has also been noted that a large set of developmental genes must be repressed in hESCs to maintain pluripotency, and their activation leads to ES cell differentiation [4]. The cytoplasmic signaling cascades controlling hESC activities are largely unknown.

Since mouse ES cells (mESCs) have received extensive attentions in the past two decades and relatively more information is available, characterization of hESC properties has been naturally conducted by referencing mESCs. Comparative analyses between hESCs and mESCs by several groups have defined common and distinct marker gene expression patterns, cellular properties and signaling mechanisms [5]–[8]. A number of cytokines, such as leukemia inhibitory factor (LIF), bone morphogenic protein 4 (BMP4), basic fibroblast growth factor (bFGF) and Wnt are known to play critical roles in regulation of mESC and/or hESC pluripotency in culture [9]–[11]. Interestingly, LIF, a most critical cytokine for culture of pluripotent mESCs, is found unnecessary for maintenance of hESC self-renewal in vitro [12]. BMP4 works in concert with LIF in promoting mESC self-renewal [13], while hESCs need the BMP antagonist, Noggin, for suppression of differentiation in vitro [11], [14]. One striking difference in intracellular signaling mechanism between hESCs and mESCs is the value of the Stat3 pathway. Stat3, which has a critical role in maintaining mESC self-renewal [15], has been found dispensable for hESCs [16], [12], [17]. However, activation of the Erk pathway leads to differentiation in both human and mouse ES cells, as expression of dominant negative mutants or use of pharmaceutical inhibitors of Mek suppressed hESC and mESC differentiation [18].

Shp2 is a cytoplasmic phospho-tyrosine phosphatase with two Src-homology 2 (SH2) domains at the NH2-terminus that modulates signal strength downstream of cytokine/growth factor receptors [19]–[21]. In particular, Shp2 has been shown to play a critical role in regulation of hematopoietic stem cell commitment and differentiation into all blood cell lineages, and somatic gain of function mutations in *PTPN11/Shp2* are implicated in leukemogenesis in human patients [22]–[26]. In this study, we have conducted a comparative analysis of Shp2 functions in mouse and human ES cells, by using combined genetics and chemical biology approaches. We established and characterized homozygous Shp2 null mutant mESC lines, and we examined the effect of Shp2 knockdown on hESC differentiation using the small RNA interference technology. By screening a chemical library, we isolated a selective Shp2 enzyme inhibitor that can suppress differentiation potential of both mESCs and hESCs. Together, our experimental results indicate that Shp2 has a conserved role in promoting differentiation of both mouse and human ES cells, via modulation of common and distinct signaling pathways.

## Materials and Methods

### Establishment of Shp2^−/−^ mESC lines

Generation of *Shp2^flox^* mice has been described previously [27]. The *Shp2^+/flox^* fertilized eggs were injected with pCMV-Cre (pBS185) plasmid DNA to generate *Shp2^+/−^* mice. The *Shp2^+/−^* mice were further crossed with UBC-GFP mice to generate *Shp2^+/−^:GFP* mice. The resultant *Shp2^+/−^:GFP* mice in C57BL/6 background were backcrossed with 129/sv wt mice for two generations. To derive mESC lines, *Shp2^+/−^: GFP* mice were intercrossed, and blastocysts (E3.5) were collected and cultured as described previously [28]. Briefly, blastocysts were collected in M2 medium, and seeded on irradiated mouse embryonic fibroblasts (MEFs) in standard mESC medium supplemented with LIF (1,000 U/ml), for growth and expansion of mESCs. Genotyping of established mESC lines was determined by PCR using primers for *wt* allele: forward: 5′-ACG TCA TGA TCC GCT GTC AG-3′, reverse: 5′-ATG GGA GGG ACA GTG CAG TG-3′; *Shp2* ^−^ allele: forward: 5′-CAG TTG CAA CTT TCT TAC CTC-3′, reverse: 5′-GCA GGA GAC TGC AGC TCA GTG ATG-3′, as described previously [27].

### ES cell culture

mESCs were maintained on irradiated primary MEFs in standard mESC culture medium. Feeder-free cultures were maintained on gelatinized tissue culture dishes. hESCs (H14, H9, H1) were cultured on irradiated MEFs or Hs27 cells in standard hESC medium − DMEM/F12 (Invitrogen) supplemented with 20% (vol/vol) Knockout™ Serum Replacement (Invitrogen), 0.1 mM 2-mercaptoethanol (Invitrogen), and 0.1 mM nonessential amino acids (Invitrogen) in the presence of bFGF (8 ng/ml, Invitrogen) or on Matrigel-coated dishes in MEF-conditioned medium, CM (R & D Systems). To quantitate proliferation, viable single mESCs were plated at 5000 cells per well in gelatin-coated 96-well plates in triplicate. Cell proliferation rates were determined by CytoQuant fluorescence assay [29], [30], and the relative proliferating rates were normalized against the cell number at 12 hrs.

### In vitro differentiation of mESCs

To assess self-renewal and differentiation, primary embryoid bodies (EBs) were formed by seeding single-cell suspension of mESCs at 10,000 cells/ml in standard mESC medium without LIF. Secondary EBs were formed by dissociating primary EBs into single cells and seeding 100,000 cells/ml in methylcellulose medium − IMDM supplemented with 15% ES-Cult™FBS, 1% methylcellulose (StemCell Technologies Inc), 2 mM L-Glutamine, 150 µM MTG, 25 mg/ml holo-transferin, 5 mg/ml ascorbic acid. Colony forming assay was performed by re-seeding cells dissociated from 1^st^ EBs onto feeder cells in the standard mESC medium (+LIF).

### Alkaline phosphatase (AP) staining and immunostainig

AP staining was performed using an alkaline phosphatase detection kit (Millipore). For immunocytochemistry, cells were fixed with 4% PFA for 30 min at room temperature. After washing with PBS, cells were treated with PBS containing 5% normal goat serum (Sigma), and 0.1% Triton X-100 for 60 min at room temperature. Then, the cells were incubated in primary antibody overnight at 4°C. Primary antibodies used include Oct3/4, Sox2, Nanog, Ssea-1, SSEA-4, BRACHYURY, GATA4, TUJ1 (Abcam). Secondary antibodies used were Alexa488-conjugated and Alexa594-conjugated IgG (Invitrogen). Nucleus was stained with DAPI. Flow cytometry was performed using BD FACSCanto analyzer with Alexa647-conjugated antibodies against Ssea-1 (Santa Cruz), Oct3/4, Sox2 (from BD pharmagen).

### Transfection and in vitro differentiation of hESCs

H14 cells were cultured 3–4 days before transfection. Short interfering RNAs specific for *PTPN11/Shp2* (and the control RNAs with scrambled sequence) were purchased from DHARMACON, as 4 oligonucleotide mixture. Sequences of the oligonucleotides are: 5′-GAACAUCACGGGCAAUUAAUU-3′; 5′-GAACACUGGUGAUUACUAUUU-3′; 5′-GAAACCAAGUGCAACAAUUUU-3′; 5′-GAAGCACAGUACCGAUUUAUU-3′. The nucleotide mixture (200 pmol) were mixed with ∼2×10^6^ H14 cells in small clumps in nucleofection solution (AMAXA, mouse ES nucleofection Kit), nucleofection was performed with program A23, and cells were slightly recovered in RPMI medium for 15 min at 37°C and then seeded on Matrigel-coated dishes in CM (R & D Systems). Unconditioned medium (UM, standard hESC medium without bFGF) was used to induce differentiation at day 2.

### Microarray analysis

Total RNA was extracted from feeder-free ES cells or EBs using RNA extraction kit (Ambion) and was reverse-transcribed. Microarray analysis was performed at the Burnham core facility by using Illumina chips.

### Reverse transcription-polymerase chain reaction (RT-PCR)

Total RNA was extracted from feeder-free ES cells using RNA extraction kit (Ambion) and was reverse-transcribed using Superscript II (Invitrogen) to generate the first-strand cDNA. Quantitative-PCR (Q-PCR) was performed with Power SYBR Q-PCR reagent (Applied Bioscience). The primers for Q-PCR are designed through Roche probe library and their sequence information is provided in Table S1.

### Molecular signaling analysis

For cytokine or growth factor stimulation, MEF cells or feeder-free mESCs/hESCs were starved in DMEM with 0.5% FBS (for MEFs and mESCs) or 0.5% SR (for hESCs) overnight (for MEFs) or for 4–6 hrs (for ES cells) prior to stimulation with LIF (1,000 U/ml, Millipore), bFGF (50 ng/ml, Invitrogen), BMP4 (25 ng/ml, R & D Systems), IGF-1 (100 ng/ml, PEPROTECH), PDGFbb (50 ng/ml, PEPROTECH). Immunoblotting analysis was performed using antibodies specific for Shp2 (c-18, Santa Cruz), Erk1/2, Stat3, Smad1 (Cell Signaling), Tubulin (Sigma), and antibodies specific for p-Erk1/2 (Thr202/Tyr204), pY-Stat3(Tyr705), p-Smad1 (Ser463/465)/Smad5 (Ser463/465)/Smad8 (Ser426/428) (Cell Signalling).

### Isolation and characterization of Shp2 inhibitors

A GST-tagged PTP domain of mouse Shp2 and Shp1 was purified from E. Coli. Various PTPs (HePTP, LypCAT) and a dual specificity phosphatase (VHR) were purified as described previously [31]. CD45 and PTP1B were purchased from BIOMOL. GST-PTP domain of Shp2 protein (50 nmol) was incubated with 1,280 compounds (10 µM each) from Lopac™ Library and 2,000 compounds (10 µM each) from Spectrum™ Library (Microsource Corporation) at room temperature for 10 min followed by addition of 1.5 mM *p*NPP to initiate the reaction. After 30 min, the reaction was terminated with 1 M NaOH, and PTPase activity was determined by measuring the absorbance at 405 nm.

### Molecular docking model of Shp2 inhibitor (DCA)

We performed molecular docking calculations for DCA using the x-ray structure of Shp2 (PDB code: 2SHP) [32]. All docking calculations were performed using FlexX module in SYBYL suite from Tripos, Inc. The x-ray structure of Shp2 reveals that the active site is a hydrophilic binding pocket and comprises of polar residues such as D61, Y62, C459, and R465. DCA enters the binding pocket (Figure S3c) and form interactions with residues that comprise the wall of the binding pocket. The 2-hydroxyl group forms a hydrogen bond with main chain amide of H426, and the carbonyl group is involved in hydrogen bonding with side chains of E361 and K366. The binding mode is further stabilized by hydrogen bonding between 8-hydroxyl group of DCA, and Q57 and D64. To look more closely, the carbonyl group in DCA is placed close to two small binding pockets lined by residues E361 and K366 (Figure S3d, left panel A). 2-hydroxy moiety is placed near a groove (Figure S3d, left panel B). This groove is lined by residues such as N37, T59, and H426. These positions could be modified to achieve optimal interactions with the binding pockets.

## Results

### Isolation and characterization of Shp2 null mutant mESC lines

**Figure 1:**
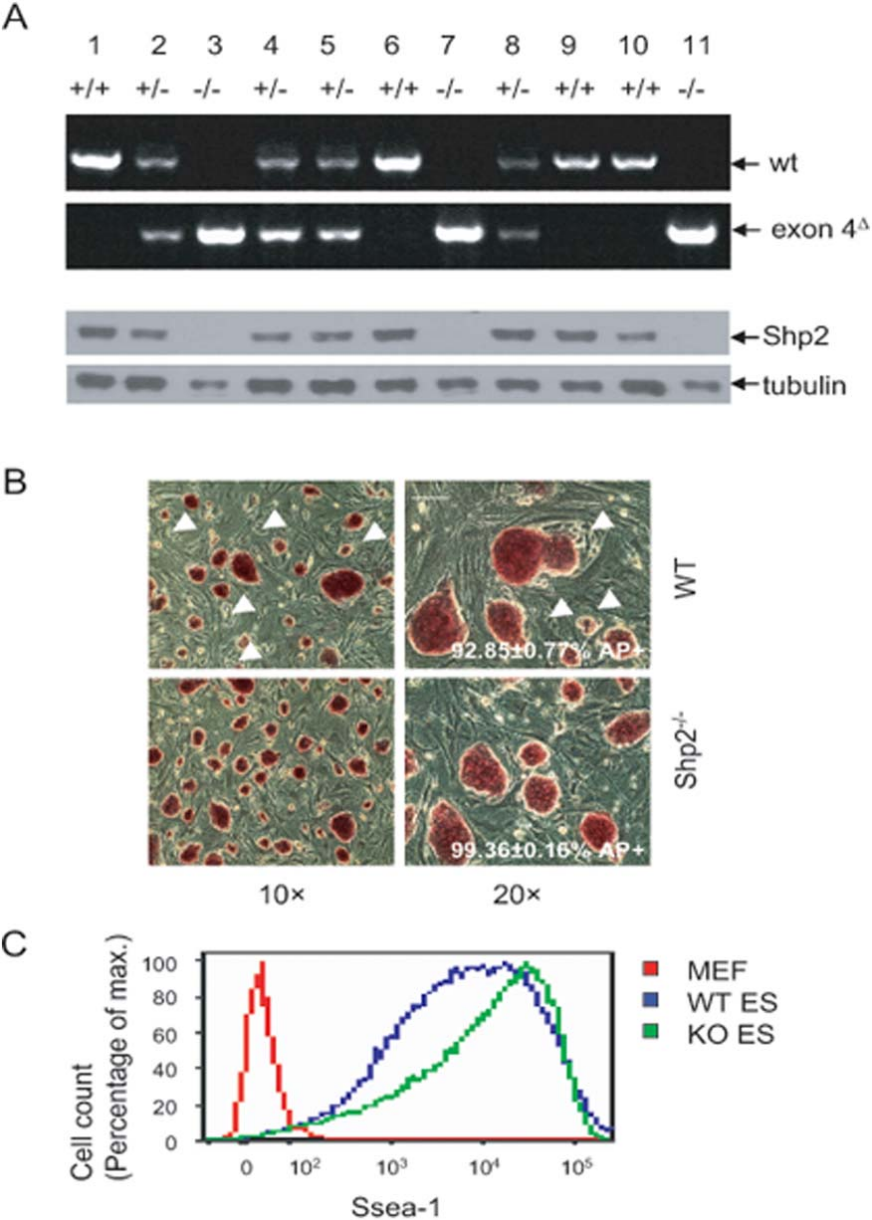
Establishment of Shp2^−/−^ mESC lines. (A) Genotyping of genomic DNA (upper panel). The wild-type (wt) and exon 4^Δ^ alleles were detected by PCR. Immunoblotting analysis for Shp2 confirmed the PCR results (lower panel), indicating that clones # 3, 7 and 11 are homozygous (*Shp2^−/−^*) mutants. (B) Alkaline phosphatase (AP) staining of *wt* and *Shp2^−/−^* mESCs cultured on feeder cells. Scale bars, 50 µm. (C) FACS analysis of Ssea-1 expression on mESCs in feeder-free culture.

In previous work, we generated homozygous mutant mES cell lines for a targeted deletion of exon 3 at the *Ptpn11/Shp2* locus [22], [33]. This in-frame deletion results in production of a truncated mutant protein Shp2^Δ46–110^, lacking amino acids 46–110 in the SH2-N domain. The homozygous mutant mESC lines for exon 3 deletion were isolated upon selection of heterozygous mutant cells with high dosages of G418, which may induce chromosome loss in ES cells. These two issues have possibly confounded the interpretation of experimental results from analysis of exon3^−/−^ cells. To unequivocally define the role of Shp2 in regulation of mESC activities, we sought to establish Shp2 null mutants. In recent experiments, we created a conditional *Shp2* knockout (*Shp2^flox^*) allele in mice by inserting two *loxP* sites into introns that flank exon 4 of *Shp2*. Deletion of exon 4 results in generation of a null mutant allele of Shp2 [27]. Following pronuclear injection of a Cre expression plasmid (pBS185) into *Shp2^flox/+^* fertilized eggs, we generated Shp2^+/−^ mice. The Shp2^+/−^ mice were further crossed with C57BL/6-Tg(UBC-GFP) 30Scha/J mice and the heterozygotes were backcrossed with 129/sv mice. We did not find any surviving Shp2^−/−^ embryos at E6.5 (data not shown), consistent with results of Yang et al [34], while homozygous Shp2 mutant embryos with exon 3 deletion die later in the uterous, around E7.5–10.5 [35]. However, we were able to isolate pre-implantation embryos at the blastocyst stage (E3.5) from pregnant Shp2^+/−^: UBC-GFP females bred with Shp2^+/−^ males, and established GFP^+^ mES cell lines from the inner cell mass as described previously [28]. Despite the early embryonic lethality due to Shp2 deletion, mESCs were successfully derived from Shp2-deficient (Exon4^−/−^) (nearly 25%), wild-type (Shp2^+/+^) and heterozygous (Shp2^+/−^) blastocysts, as detected by PCR genotyping (Figure 1A, upper panel), and ablation of Shp2 was confirmed by immunoblotting (Figure 1A, lower panel). *Shp2^−/−^* mESCs displayed decreased proliferation and similar survival rates as compared to *wt* mESCs (Figure S1A, S1B).

### Shp2^−/−^ mESCs exhibit reduced differentiation capacity with improved self-renewal potential

**Figure 2:**
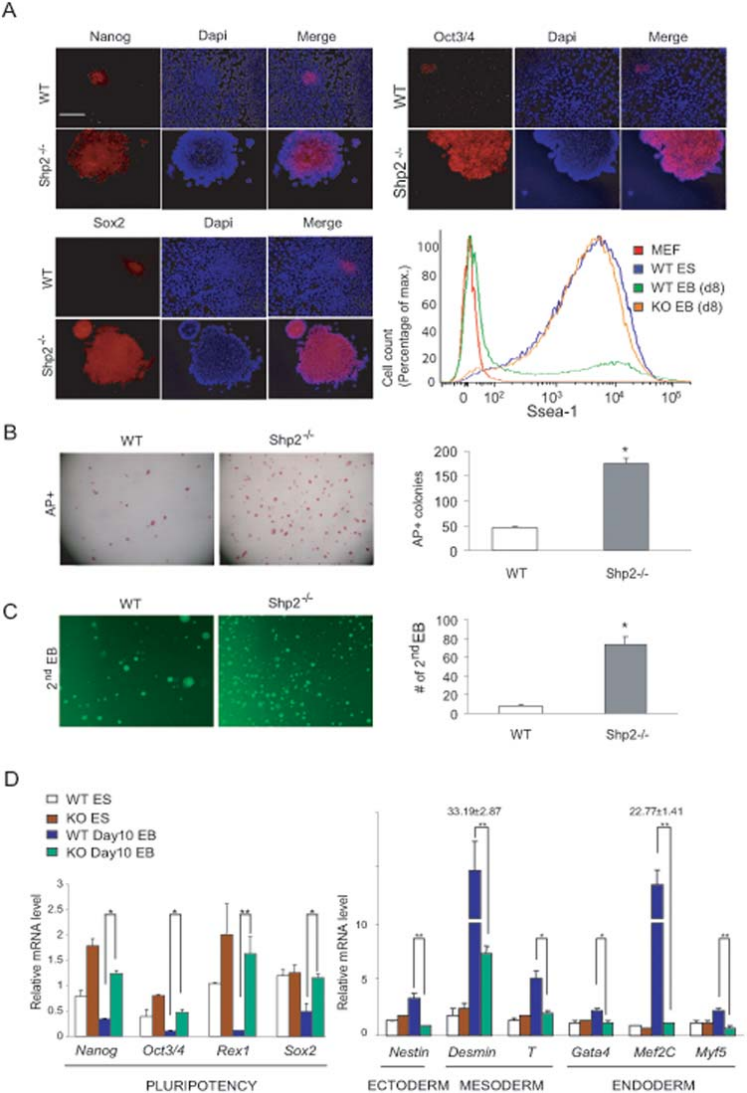
Shp2^−/−^ mES cells exhibit reduced differentiation capacity with improved self-renewal potential. (A) Immunostaining for Nanog, Oct3/4, Sox2, and flow cytometry for Ssea-1 in primary (1^st^) EBs cultured in LIF-free suspension medium for 8 days. Scale bars, 10 µm. (B) 1^st^ EBs were dissociated into single cells and re-seeded at the density of 1×10^6^ cells/ml onto feeder cells and cultured in ES cell standard medium (+LIF) for colony forming assay. The cells were fixed and AP-stained. The AP-positive cells were counted under a bright field microscope. Scale bars, 200 µm. (C) 1^st^ EBs were dissociated into single cells and re-seeded at the density of 1×10^6^ cells/ml in the same medium as 1^st^ EBs for formation of secondary (2^nd^) EBs. Scale bars, 200 µm. The number of 2^nd^ EBs was counted under a bright field microscope. (D) Total RNA was extracted from mESCs (day 0) and differentiating EBs at day10, and qRT-PCR was performed, and the relative values were normalized by *CPH*. Relative value changes of Shp2^−/−^ over wt mESCs were indicated (n>3, * *P*<0.05, ** *P*<0.01).

When cultured in standard mESC medium, all of the *Shp2^−/−^* mESC clones contained densely packed cells, with smooth outline and no differentiated cells at the edges. The *Shp2^−/−^* mESC colonies were positive for alkaline phosphatase (AP) staining after 20 passages (Figure 1B). However, the *wt* mES cells, like other mESC lines available, showed approximately 5–10% spontaneous differentiation under the same culture condition. Shp2-deficient mESCs expressed high levels of Ssea-1 as determined by FACS analysis (Figure 1C). Embryoid body (EB) formation assay was performed to evaluate mESC differentiation potential. After 8-day incubation in differentiation medium, the expression of Nanog, Oct4, and Sox2 remained high in Shp2-deficient EB cells, in contrast to their dramatically decreased expression levels detected only in the core area of *wt* EBs (Figure 2A). Flow cytometry demonstrated sustained expression of Ssea-1 on *Shp2^−/−^* EB cells at levels comparable to that of undifferentiated ES cells, while *wt* EB cells had dramatically declined Ssea-1 expression (Figure 2A). Furthermore, dissociated primary *Shp2^−/−^* EB cells can form ESC-like AP^+^ colonies much more efficiently than isolated *wt* EB cells, when seeded on feeder cell layer and cultured in standard mESC culture medium (Figure 2B). The secondary EB formation efficiency of isolated primary *Shp2^−/−^* EB cells was 10 fold higher than *wt* EB cells (Figure 2C). Together, these data suggest that ablation of Shp2 resulted in inhibition of mESC differentiation and improved maintenance of pluripotency.

### Shp2 deletion suppresses mESC differentiation into all three germ layer cell lineages

Microarray analysis data collected at different time points (Figure S2A) show dramatically impaired differentiation capacity of Shp2−/− mESCs. The overall gene expression profiles were very similar in Shp2-deficient mESCs examined at 0, 2 and 8 days of differentiation, in contrast to wt ES cells that exhibited dramatically different expression patterns during this time period. Sustained expression of pluripotent ESC marker genes (Nanog, Oct3/4, Rex1 and Sox2) in Shp2 mutant cells were confirmed by RT-PCR (Figure S2B), immunoblotting (Figure S2C), and quantitative real time RT-PCR (qRT-PCR) analysis (Figure 2D). In contrast, significantly lower expression was detected in *Shp2^−/−^* than in *wt* EB cells at day 10, for genes that are predominantly expressed in various differentiated cell lineages, such as *Nestin* (in ectoderm), *Brachyury (T)* and *Desmin* (in mesoderm), and *Gata4*, *Myf5* and *Mef2C* (in endoderm) (Figure 2D). In addition, microarray analysis also detected significantly lower expression of *Sox17*, *Sox 7* and *Foxa2* in *Shp2^−/−^* than in *wt* EB cells at day 8 (Figure S2A). Therefore, Shp2 is required for intracellular signaling controlling exit of pluripotent mESCs for differentiation into all three germ layers.

### Shp2 knockdown leads to impaired hESC differentiation

**Figure 3:**
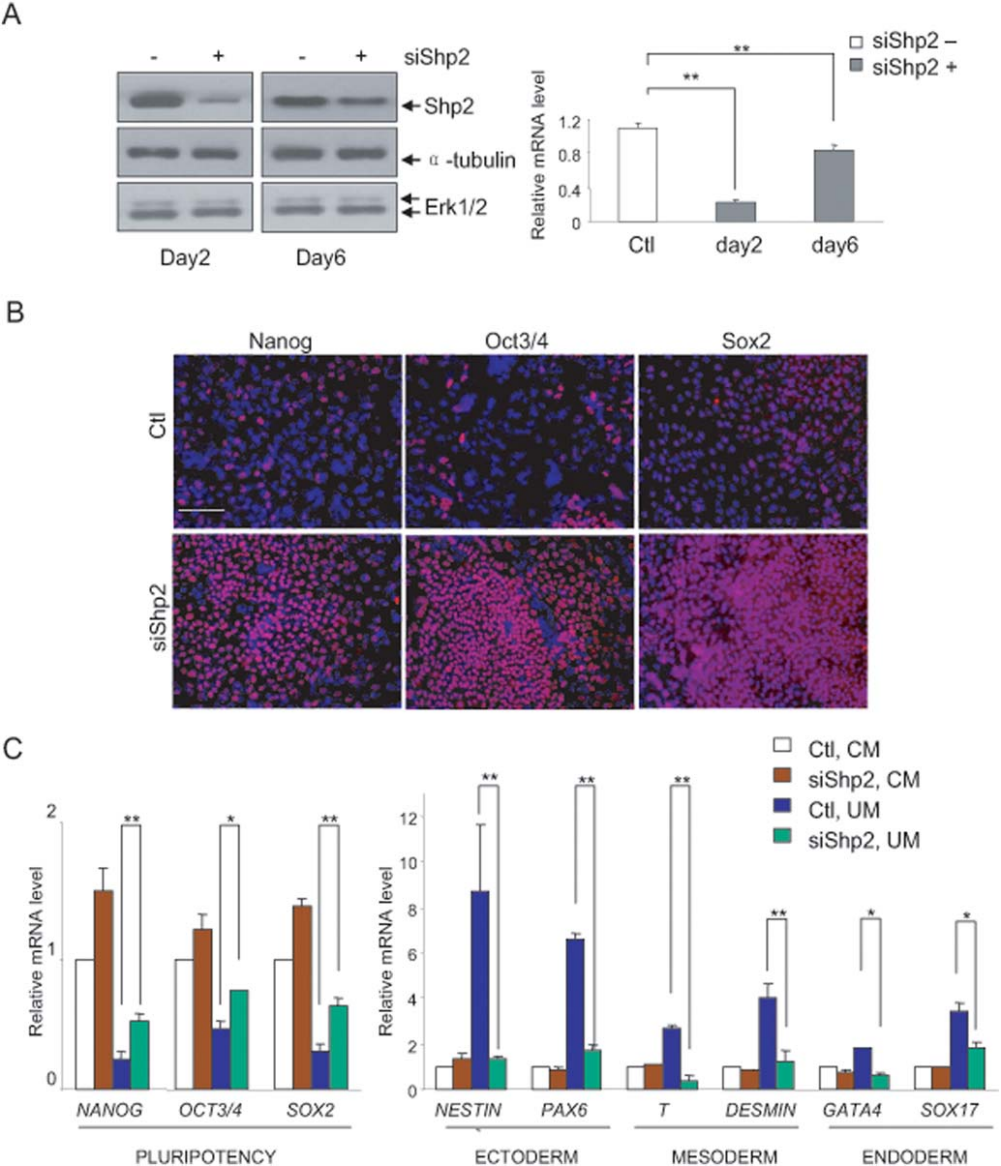
Shp2 knockdown in hESCs leads to impaired differentiation. (A) Immunoblotting and qRT-PCR indicate efficient Shp2 knockdown by 80% in hESCs. H14 cells were transfected with Shp2-specific (+siShp2) or non-specific siRNA (−siShp2), and cells were cultured on matrigel-coated dishes in CM for 2 days, total cell lysates were immunoblotted with the indicated antibodies or total RNAs were extracted for qRT-PCR (n = 3, ** *P*<0.01). (B) Immunostaining of NANOG, OCT3/4, SOX2 in hESCs. After transfection, H14 cells were cultured in CM for 2 days, then the medium were changed to UM to induce differentiation for additional 6 days. Scale bars, 10 µm. (C) Total RNAs were extracted from hESCs cultured in CM or UM, qRT-PCR was performed, and the relative values of Shp2 knockdown over control hESCs were indicated (n = 3, * *P*<0.05, ** *P*<0.01).

We then asked whether Shp2 plays a similar role in control of hESC differentiation, and used small RNA interference to knockdown expression of *PTPN11/Shp2* in hESCs. Transfection of specific siRNA resulted in downregulation of Shp2 expression by 80% in hESCs, lasting for one week (Figure 3A). Shp2 knockdown resulted in increased expression of NANOG, OCT3/4 and SOX2 in H14 hES cells detected by immunostaining (Figure 3B), and similar results were obtained for H1 and H9 hES cells (data not shown). Consistently, qRT-PCR analysis demonstrated that Shp2 knockdown hESCs expressed higher levels of *NANOG*, *OCT3/4* and *SOX2* than control cells, when cultured in conditioned medium, CM (for maintenance) or un-conditioned medium, UM (favoring differentiation) (Figure 3C). hESC differentiation was determined by measuring expression levels of *NESTIN* and *PAX6* for ectoderm, *T* and *DESMINE* for mesoderm and *GATA4* and *SOX17* for endoderm. Under CM culture condition, similar low basal expression levels of these differentiated cell markers were detected between control and Shp2 knockdown hESCs (Figure 3C). However, after 6-day incubation in UM, control hESCs differentiated rapidly with marked increase in expression of genes that are preferentially detected in three germ layer cell lineages, while Shp2 knockdown resulted in significantly impaired expression of these differentiated cell marker genes (Figure 3C). Thus, the Shp2 function in promoting initial differentiation is conserved between mouse and human ES cells.

### Shp2 modulates common and distinct pathways in mES and hES cells

**Figure 4:**
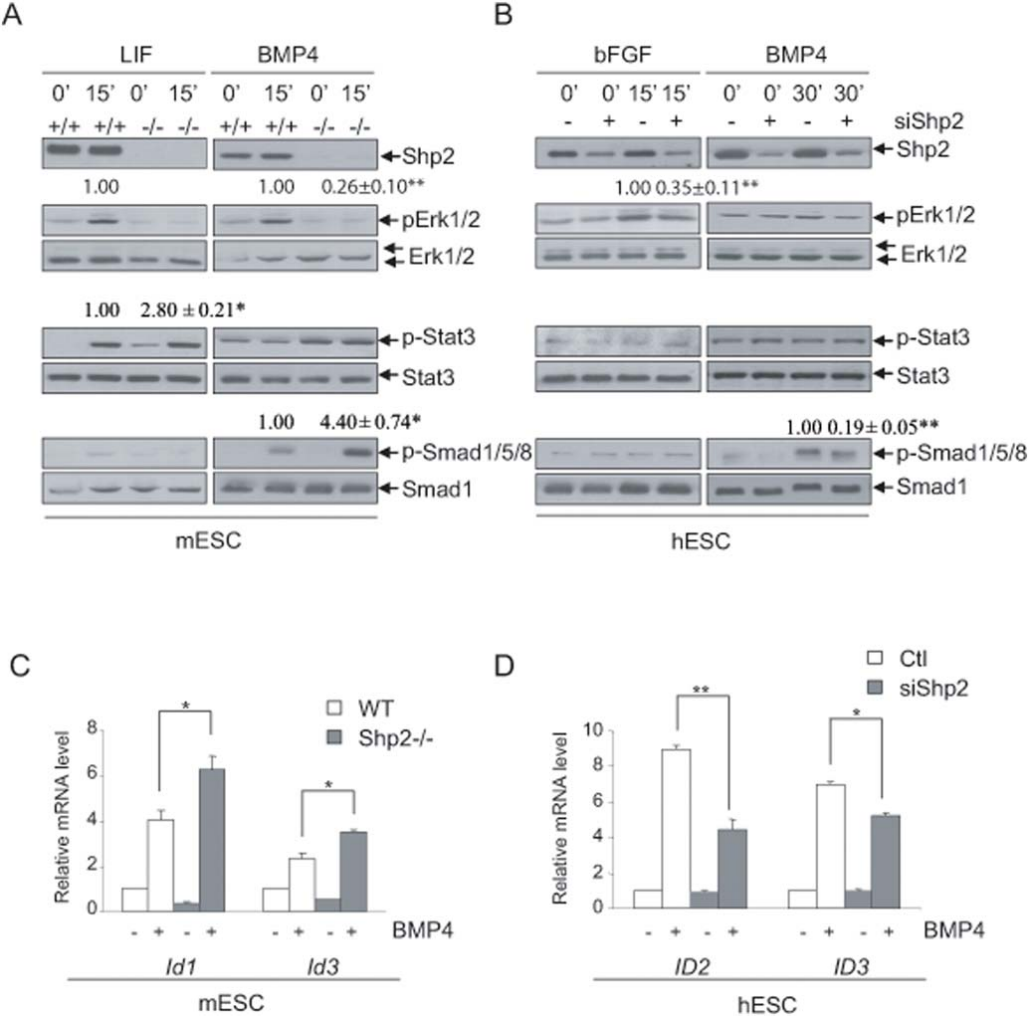
Shp2 modulates various signaling pathways in mES and hES cells. (A) LIF/BMP4 signaling in mESCs. mESCs were stimulated by LIF (1,000 units/ml) or BMP4 (25 ng/ml) for 15 mins after overnight starvation. Total cell lysates were immunoblotted with the indicated antibodies. (B) bFGF/BMP4 signaling in hESCs (H14). The indicated antibodies were used to analyze proteins in hESCs stimulated by bFGF (50 ng/ml, 15 mins) or BMP4 (25 ng/ml, 30 min) after 6-hour starvation. (C, D) mESCs (left) or hESCs (right) were treated with BMP4 for 60 mins and total RNAs were analyzed for *Id* expression levels (Mean±SEM, n = 3). (a, c, e) mESCs; (b, d, f) hESCs.

As the Erk, Stat3 and Smad pathways have been implicated in regulation of ESC self-renewal and differentiation [15], [13], [11], [18], we investigated how Shp2 modulates these signaling events in mES and hES cells. LIF, BMP4 and bFGF potently induced Erk1/2 activation in *wt* mESCs, while p-Erk signals were decreased or blunted in *Shp2^−/−^* mESCs (Figure 4A). In contrast, basal and LIF-stimulated pY-Stat3 signals were enhanced in *Shp2^−/−^* mESCs, compared to that in *wt* cells (Figure 4A). These results suggest a bi-directional regulation by Shp2 of Erk and Stat3 pathways in promoting mESC differentiation, similar to the observation made in neural stem cells [30]. Consistently, p-Erk signals were decreased in Shp2 knockdown hESCs following treatment with bFGF, but not BMP4 (Figure 4B). Therefore, Shp2 has a conserved role in promoting signaling through Erk in both mESCs and hESCs. However bFGF, BMP4 or LIF did not elicit a robust induction of pY-Stat3 signal in hESCs (Figure 4A and data not shown), reinforcing the notion that the LIF-Stat3 pathway, although critical for mESC self-renewal [36], [15], is dispensable in hESCs [16], [12], [17].

### Shp2 modulates the BMP-Smad pathway bi-directionally in mouse and human ES cells

Interestingly, Shp2 ablation in mESCs resulted in increased p-Smad1/5/8 signals following BMP4 treatment, but not LIF stimulation (Figure 4A). In contrast, decreased levels of p-Smad1/5/8 were detected in Shp2 knockdown hESCs following BMP4 (but not bFGF) stimulation, compared to that in control cells (Figure 4B). These results suggest opposite effects of Shp2 on BMP4-Smad signaling in mESCs and hESCs. To corroborate and extend these observations, we performed qRT-PCR analysis of expression profiles for genes downstream of the BMP4-Smad pathway. Results in Figure 4C and 4D demonstrate significantly increased BMP4 induction of *Id1* and *Id3* expression in Shp2^−/−^ mESCs compared to wt cells, while BMP4-stimulated *ID2* and *ID3* expression levels were lower in Shp2 knockdown than in control hESCs. Notably, BMP4-Smad signaling has been shown to work cooperatively with LIF-Stat3 pathway in supporting mESC self-renewal [13]. However, BMP4 can induce trophoblast differentiation in hESCs and a BMP antagonist, Noggin, acts to support hESC self-renewal in coordination with bFGF [11]. Thus, Shp2 may act to orchestrate hESC/mESC differentiation at least in part through bi-directional regulation of BMP4-Smad signaling in the two types of stem cells. Modulation of BMP4-Smad signal strength by Shp2 may involve direct and indirect mechanisms, cross-talks between Erk, Stat3 and Smad pathways have been documented in the literature [37].

### Shp2 inhibitors attenuate both mESC and hESC differentiation

The conserved function of Shp2 in mESC/hESC differentiation prompted us to search for Shp2 inhibitors. By screening the Lopac™ and Spectrum™ library (Microsource Corporation), we identified 56 compounds that showed potent inhibitory effect on Shp2 catalytic activity, and three of these compounds exhibited highly selective inhibition on Shp2 (Figure S3A). In this study, we focused on 7-deshydroxypyrogallin-4-carboxylic acid (DCA) (Figure S3B), with an IC_50_ on Shp2 as 2.1 µM, but much less active on several other tyrosine phosphatases tested, including HePTP, LypCAT, CD45, PTP1B and a dual specificity phosphatase VHR (Figure S3C). A similar inhibitory effect on Shp1 (IC_50_: 2.3 µM), a close relative of Shp2, does not affect our experimental results, since Shp1 is not expressed in ES cells. Ki determination suggests that DCA may function as a mixed type (competitive and/or uncompetitive) inhibitor with low value (2.6 µM). A computer-based docking model shows that DCA can access and enter the pocket located in the PTP domain (Figure S4A, S4B). DCA suppressed growth factor-stimulated Erk activity in cells, as evaluated by both kinase assay using MBP as substrate and immunoblotting against p-Erk1/2 (Figure S3D), consistent with a positive role of Shp2 in mediating Erk activation by growth factors [19], [20]. Of Note, DCA did not affect IGF-1 stimulated Erk activation in *Shp2^−/−^* mESCs (Figure S5A), indicating specificity of the compound toward Shp2. Furthermore, DCA treatment had no effect on IGF-1-stimulated Akt activity in *wt* and *Shp2^−/−^* mESCs, ruling out the possibility of non-specific DCA interference in cell signaling (Figure S5A). DCA at high concentration (100 µM) showed inhibitory effect on ESC proliferation, with no toxicity to both mouse and human ES cells at low concentrations (Figure S5B). Furthermore, mES cell proliferation rate can be restored following removal of the compound from culture medium, suggesting its reversible effect (Figure S5C).

**Figure 5:**
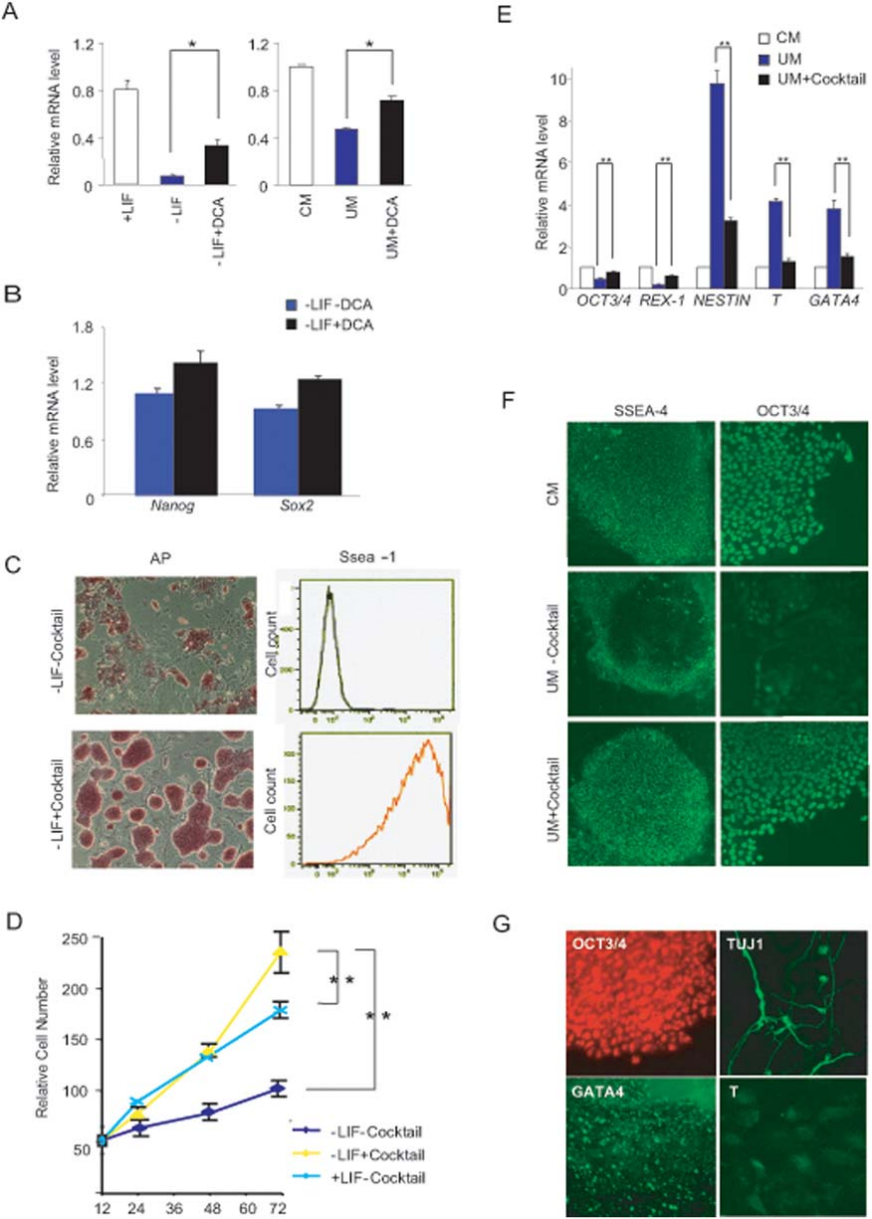
Chemical Inhibitors suppress mESC and hESC differentiation. (A) DCA partially inhibits differentiation in both mESCs (left panel) and hESCs (right panel) monitored by qRT-PCR analysis of *Oct3/4* expression. (B) qRT-PCR analysis of pluripotent cell markers *Nanog* and *Sox2*. mESCs were incubated in feeder-free and LIF-free medium in the absence or presence of 50 µM DCA for 6 days and total RNAs were extracted for analysis (Mean±SEM, n = 3). (C) AP staining (left panel) and Ssea-1 flow cytometry (right panel) of mESCs treated with or without cocktail inhibitors. Scale bars, 50 µm. (D) mESCs cultured in feeder-free and LIF-free medium with cocktail inhibitors exhibited comparable proliferation rate as cells cultured in standard mESC medium (+LIF). (E) QRT-PCR analysis of pluripotent cell markers *OCT3/4* and *REX-1*, or differentiated cell markers *NESTIN*, *T* and *GATA4*. hESCs were incubated in CM, or UM in the absence or presence of cocktail inhibitors as indicated (25 µM DCA, 25 µM PD and 0.5 µM Bio) for 6 days (Mean±SEM, n = 3). (F) Morphological analysis of hESCs performed by immunostaining of OCT3/4, and SSEA-4. Scale bar, 10 µm. (G) Removal of cocktail inhibitors restored differentiation capacity. hESC cells maintained with the cocktail inhibitors were incubated in UM without inhibitors for 8 days and allowed for differentiation under standard procedures. Cells were immunostained for TUJ1^+^ neurons, GATA4^+^ endoderm cell lineage or T^+^ mesoderm cell lineage, and hES cells incubated in CM were immunostained for OCT3/4. Scale bar, 5 µm.

We analyzed DCA effect on mESC and hESC differentiation. Supplement of DCA in un-conditioned medium partially suppressed mESC and hESC differentiation, as monitored by *Oct3/4*, *Nanog* and *Sox2* expression (Figure 5A, 5B). DCA at low dosages was not sufficient to maintain ESC pluripotency when added into the culture medium, while high dosages of DCA were toxic to ES cells. Chemical modification of DCA may improve its efficacy and specificity and we will also expand our screening of chemical libraries to search for more efficient Shp2 inhibitors. In this study, we sought to compose a cocktail consisting of DCA and two other compounds, PD98059 (Mek inhibitor) and BIO (Gsk3 inhibitor), which were shown to partially inhibit mESC/hESC differentiation [38], [18]. Combination of these three compounds at low concentrations efficiently suppressed mESC differentiation in LIF- and feeder-free culture medium without significant impact on cell proliferation rate (Figure 5C, 5D). mESCs maintained with the cocktail inhibitors expressed high levels of AP and Ssea-1 for up to 20 passages (Figure 5C). We also observed a similar effect of the cocktail inhibitors in suppressing hESC differentiation. qRT-PCR analysis demonstrated increased expression levels of pluripotent stem cell markers, *OCT3/4* and *REX-1*, accompanied by decreased expression of differentiated cell markers *NESTIN*, *T* and *GATA4* (Figure 5E). hESCs were passaged in feeder-free medium supplemented with cocktail inhibitors for at least three generations without obvious differentiation (Figure 5F) and restored differentiation into three cell lineages following drug removal (Figure 5G), suggesting reversible effect of the inhibitors. Therefore, this chemical biology approach renders support to the genetic data that suggest a conserved role of Shp2 in control of mESC and hESC differentiation.

## Discussion

The intracellular signaling mechanism for the switch between self-renewal and differentiation of ES cells is a central and unanswered question in stem cell biology, and the initiation of ESC differentiation is apparently determined by multiple pathways working in concert. This complexity requires a modulator(s) that fine-tunes signals coordinately in negative or positive fashion, although little is known in this regard. We demonstrate here that Shp2 acts as a negative or positive regulator of signaling events controlling self-renewal and differentiation of mESCs and hESCs.

In this study, we have taken three different experimental approaches to determine Shp2 functions in human and mouse ES cell differentiation. First, we established homozygous mutant mES cell lines with a targeted deletion of exon 4 at the *Shp2* locus. Shp2-deficient mES cells displayed dramatically impaired capacity of differentiation into all three germ layer cell lineages, accompanied by improved self-renewal potential. These results clearly define a biological function of Shp2 in control of mouse ES cell pluripotency and differentiation. Similar results were obtained from Shp2 mutant (exon 3^−/−^) mES cells in our previous experiments [33], [25], supporting the notion that targeted deletion of exon 3 at the *Ptpn11/Shp2* locus created a loss of function mutant Shp2 molecule. Second, to extend the functional analysis of Shp2 from mouse to human ES cells, we have successfully used the siRNA approach to knockdown Shp2 expression in hESCs, unveiling a similar role of Shp2 in promoting human ES cell differentiation. Thus, the Shp2 function in promoting ES cell differentiation is conserved between mESCs and hESCs, despite a body of literature documenting differences existing between the two types of ES cells [5]–[8]. Third, a small molecule inhibitor of Shp2 enzyme has been identified that partially inhibits mouse and human ES cell differentiation at low dosages. This chemical biology experiment not only renders strong support to our gene knockout and knockdown experiments, but also suggests that development of specific Shp2 inhibitors can provide useful experimental tools for amplification of hESCs in vitro for molecular analysis and biological characterization.

Our results suggest different molecular mechanisms underlying the concerved function of Shp2 in regulation of mouse and human ES cell differentiation. In mouse ESCs, Shp2 participates in modulation of LIF signals by promoting the Erk pathway and suppressing the Stat3 signal. Upon LIF stimulation, Shp2 is physically recruited to gp130, the signaling component of LIF receptor, and acts to regulate the LIF signal [39], [25]. Bi-directional regulation of Erk and Stat3 pathways by Shp2 appears to be conserved in embryonic and adult stem cells as well as in differentiated cell types. In previous work, we have found opposite effects of Shp2 deletion on Erk and Stat3 activation in coordinated regulation of neural stem cell proliferation and differentiation into neuronal/glial cell lineages during brain development [30], and also in control of energy balance by leptin in hypothalamic neurons of adult brain [27]. In addition, we detected a similar fashion for Shp2 regulation of Erk and Stat3 activation in hepatocytes during liver regeneration and epithelial cells in mammary gland development and involution [40], [41]. Both the decreased Erk activity and enhanced Stat3 signals may lead to suppression of mES cell differentiation and improved self-renewal. Attenuation of Erk signal may also be responsible for the decreased proliferation rate observed in Shp2^−/−^ mESCs and Shp2 knockdown hESCs as well as in mESCs/hESCs treated with Shp2 inhibitors. Activation of the Stat3 pathway may lead to different physiological consequences in various cell types. The LIF-Stat3 signaling appears to be required for self-renewal in mESCs [36], [15], while activation of Stat3 promotes glial cell differentiation from neural stem/progenitor cells during cell fate specification in the brain [42]. On the other hand, Stat3 is required for timely initiation of physiological epithelial cell apoptosis during mammary gland involution [43]. Consistent with the literature [16], [12], [17], we did not detect Stat3 activation signals in hESCs under a variety of growth factor/cytokine stimulation, supporting the theory that the Stat3 pathway is not required for hESC self-renewal.

One interesting observation made in this study is the bi-directional regulation of BMP4-Smad signaling by Shp2 in mouse and human ES cells. Genetic ablation of Shp2 in mouse ES cells resulted in enhanced p-Smad1/5/8 signals in response to BMP4 stimulation and, consistently, increased expression of *Id1* and *Id3*, target genes downstream of the BMP4-Smad pathway, was detected in Shp2^−/−^ mESCs compared to wild-type cells. In contrast, Shp2 knockdown in hESCs leads to impaired p-Smad1/5/8 levels following BMP4 treatment. Consistent to this observation is the decreased induction of *ID2* and *ID3* gene expression by BMP4 in Shp2 knockdown hESCs. These results suggest a cell context-dependent manner for Shp2 regulation of the BMP4-Smad pathway in hESCs and mESCs. Interestingly, this bi-directional modulation of Smad signaling can lead to the same consequence of differentiation suppression in human and mouse ES cells. BMP4-Smad signaling has been shown to work cooperatively with the LIF-Stat3 pathway in supporting mouse ES cell self-renewal [13]. However, BMP4 can induce trophoblast differentiation in human ES cells , and Noggin, a BMP antagonist, has been shown to support hESC self-renewal in concert with bFGF [11]. It is unclear yet how Shp2 regulates the BMP4-Smad pathway, which may involve direct and indirect mechanisms. For example, Shp2 may influence Smad signaling strength via regulation of Erk and Stat pathways, since cross-talks between Erk, Stat and Smad pathways have been reported previously by several groups [37].

Deletion of Shp2 also resulted in reduced mESC proliferation, suggesting a role of Shp2 in mitogenic signaling. Consistently, we observed reduced proliferation rate of hESCs when Shp2 expression is downregulated by siRNA, and supplement of Shp2 inhibitor DCA leads to reduced cell proliferation of both hESCs and mESCs. However, the reduced differentiation capacity of Shp2-deficient ES cells is unlikely due to decreased proliferation rate. In contrast, the primary and secondary EB formation assay detected dramatically increased self-renewing proliferation of Shp2^−/−^ mES cells, as compared to wt cells. Furthermore, molecular signaling analysis strongly suggests that Shp2 ablation suppressed pathways favoring differentiation, while enhancing signals leading to self-renewal. The cross-talk and balance of proliferation, self-renewal and differentiation signals modulated/coordinated by Shp2 contribute to the switch between pluripotency and differentiation of ES cells.

We have identified a potent and selective Shp2 inhibitor by screening a small chemical library. The specificity of Shp2 inhibition by DCA has been tested and confirmed by a number of experiments in this study: a) inhibition of purified Shp2 was examined against a list of other PTPs; b) DCA was shown to suppress growth factor-stimulated Erk activation; c) DCA was found to have no effect on Shp2^−/−^ cells. As expected, the inhibitors can suppress growth factor-stimulated Erk activation and also cell proliferation. Importantly, DCA exhibits similar inhibitory effects on mouse and human ES cell differentiation. In recent experiments, several groups have shown similar effects with Mek inhibitor and GSK3 inhibitor on mouse or human ES cells [38], [18]. One concern with small molecule inhibitors is often associated with their toxicity that limits their value in their use for long-term hESC culture. We have used an inhibitor cocktail and our results show that in combination, these molecules can be used at much lower concentrations to reach a similar biological effect. Further chemical modification of the isolated small molecule in order to increase specificity and reduce toxicity will increase the application value.

In summary, multiple pathways, including Erk, Stat3 and Smad, have been shown to participate in cellular decisions for ESC self-renewal or differentiation; this study identifies Shp2 as a critical player orchestrating these pathways in programming initial differentiation of human and mouse ES cells.

## Supporting Information

Figure S1 Growth, survival and differentiation of Shp2−/− mES cells. (A) Proliferation rate. mESCs were seeded on gelatin-coated plates at 50,000 cells/ml in the absence of feeder cells and cell numbers were determined by CytoQuant fluorescence assay at 12, 24, 36, 48, 72 hrs after seeding. The cell numbers were normalized against the value at 12 hrs (Mean±SEM, n = 4). (B) Immunostaining of BrdU+ cells (red) co-labeled with DAPI (blue) after 4 hr incubation (upper panel). Annexin V staining for cell apoptosis (lower panel). Scale bar, 20 µm.
(1.73 MB TIF)

Figure S2 Gene expression profiles during mESC differentiation. (A) Total RNAs were extracted from three seperate mESC lines for both WT and Shp2−/− during differentiation at various time points as indicated. The microarray data collected from the triplacate samples were grouped and analysed by GeneSpring GX Software (Agilent Technologies). (B) Total RNA samples collected at different time points during differentiation were subjected to RT-PCR for Oct3/4 detection. (C) The whole cell lysates collected at different time points during differentiation were subjected to immunoblotting by Oct3/4 and Nanog antibodies.
(2.20 MB TIF)

Figure S3 Isolation of Shp2 inhibitors. (A) Screening procedure of chemical library. (B) The chemical structure of DCA. (C) IC50 values of DCA against various PTPs. (D) DCA inhibits Erk phosphorylation and activity in MEFs. Overnight-starved MEFs were treated with increasing dosages of DCA: 5, 20, 100 µM for 1 hr followed by stimulation with PDGFbb (50 ng/ml, 5 min). pErk1/2 was detected in whole cell lysates. Erk1/2 was immunoprecipitated from cell lysates and kinase activity was measured using myelin basic protein (MBP) as substrate (with [32P]ATP). After autoradiography, the membrane was used for immunoblotting analysis of Erk1/2. Total cell lysates were also immunoblotted as indicated.
(2.01 MB TIF)

Figure S4 Computerized model for DCA interaction with Shp2. (A) A computerized mode of DCA binding to the active site of Shp2. (B) A: zoomed in view of DCA in the active site of Shp2 shows the carboxylic acid group which is placed close to small binding pockets (indicated by a white arrow). B: the 2-hydroxyl group is situated near a groove, indicated by a white arrow.
(2.01 MB TIF)

Figure S5 Biological activity of Shp2 inhibitor (DCA). (A) Specificity of DCA in mESCs. Overnight-starved mESCs were treated with 50 µM DCA for 1 hr followed by stimulation with IGF-1 (100 ng/ml) for 10 min. Total cell lysates were immunoblotted with the indicated antibodies. (B) CytoQuant fluorescence assay for proliferation of mESCs treated with DMSO or DCA (20 µM or 100 µM). The data shown are means±SEM, n = 4). (C) Reversible effect of DCA on mESCs proliferation. After pretreatment with 100 µM DCA for 36 hrs, mESCs were washed with PBS 3 times and then cultured in the same medium but with DMSO, up to 72 hrs (Means±SEM, n = 4).
(2.35 MB TIF)

Table S1 DNA sequences of primers used for qRT-PCR.
(0.06 MB DOC)
