# Supplementary figures and images for: A Conserved Mechanism for Control of Human and Mouse Embryonic Stem Cell Pluripotency and Differentiation by Shp2 Tyrosine Phosphatase
Source: PLoS One. 2009 Mar 17;4(3):e4914. doi: 10.1371/journal.pone.0004914 (PMC2655646; doi:10.1371/journal.pone.0004914)

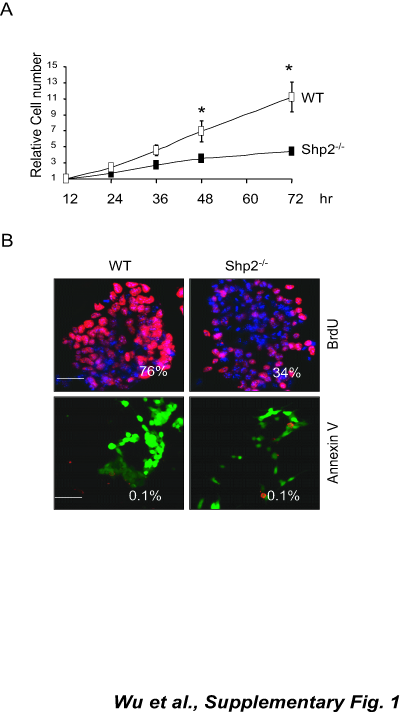

Supplement: Figure S1 — Growth, survival and differentiation of Shp2−/− mES cells. (A) Proliferation rate. mESCs were seeded on gelatin-coated plates at 50,000 cells/ml in the absence of feeder cells and cell numbers were determined by CytoQuant fluorescence assay at 12, 24, 36, 48, 72 hrs after seeding. The cell numbers were normalized against the value at 12 hrs (Mean±SEM, n = 4). (B) Immunostaining of BrdU+ cells (red) co-labeled with DAPI (blue) after 4 hr incubation (upper panel). Annexin V staining for cell apoptosis (lower panel). Scale bar, 20 µm. (1.73 MB TIF) [file pone.0004914.s001.tif]

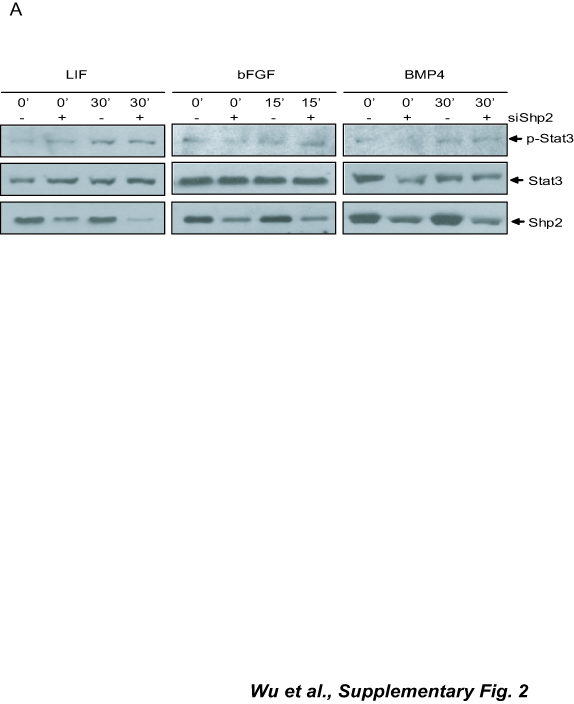

Supplement: Figure S2 — Gene expression profiles during mESC differentiation. (A) Total RNAs were extracted from three seperate mESC lines for both WT and Shp2−/− during differentiation at various time points as indicated. The microarray data collected from the triplacate samples were grouped and analysed by GeneSpring GX Software (Agilent Technologies). (B) Total RNA samples collected at different time points during differentiation were subjected to RT-PCR for Oct3/4 detection. (C) The whole cell lysates collected at different time points during differentiation were subjected to immunoblotting by Oct3/4 and Nanog antibodies. (2.20 MB TIF) [file pone.0004914.s002.tif]

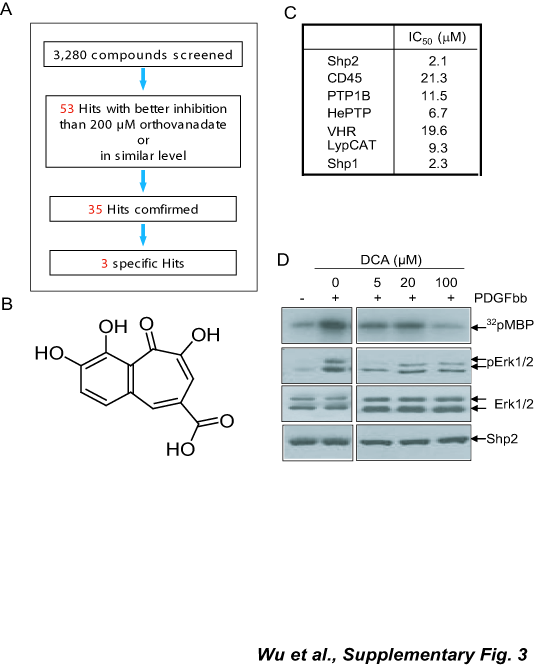

Supplement: Figure S3 — Isolation of Shp2 inhibitors. (A) Screening procedure of chemical library. (B) The chemical structure of DCA. (C) IC50 values of DCA against various PTPs. (D) DCA inhibits Erk phosphorylation and activity in MEFs. Overnight-starved MEFs were treated with increasing dosages of DCA: 5, 20, 100 µM for 1 hr followed by stimulation with PDGFbb (50 ng/ml, 5 min). pErk1/2 was detected in whole cell lysates. Erk1/2 was immunoprecipitated from cell lysates and kinase activity was measured using myelin basic protein (MBP) as substrate (with [32P]ATP). After autoradiography, the membrane was used for immunoblotting analysis of Erk1/2. Total cell lysates were also immunoblotted as indicated. (2.01 MB TIF) [file pone.0004914.s003.tif]

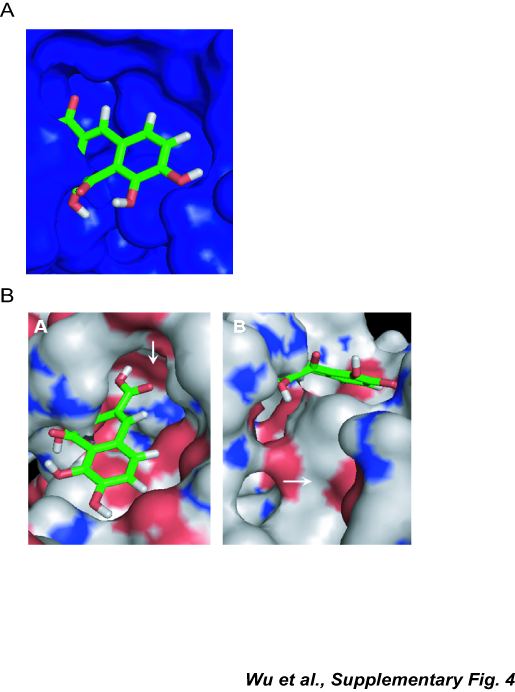

Supplement: Figure S4 — Computerized model for DCA interaction with Shp2. (A) A computerized mode of DCA binding to the active site of Shp2. (B) A: zoomed in view of DCA in the active site of Shp2 shows the carboxylic acid group which is placed close to small binding pockets (indicated by a white arrow). B: the 2-hydroxyl group is situated near a groove, indicated by a white arrow. (2.01 MB TIF) [file pone.0004914.s004.tif]

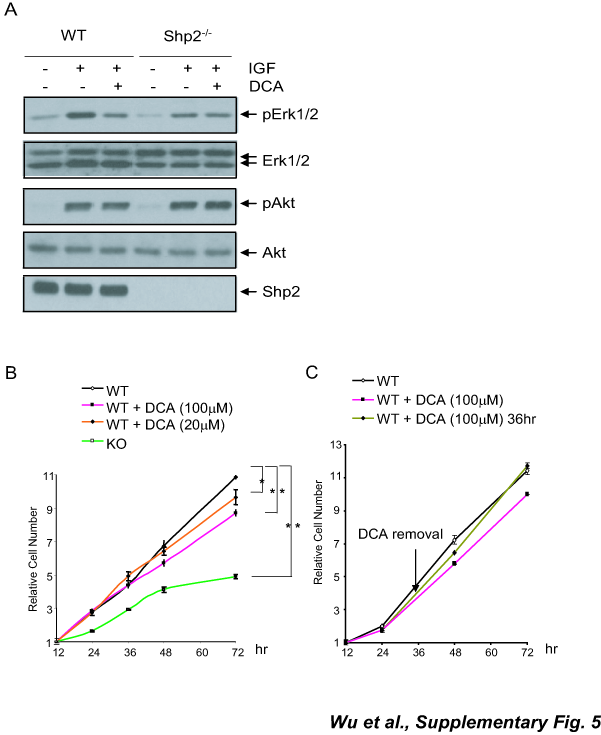

Supplement: Figure S5 — Biological activity of Shp2 inhibitor (DCA). (A) Specificity of DCA in mESCs. Overnight-starved mESCs were treated with 50 µM DCA for 1 hr followed by stimulation with IGF-1 (100 ng/ml) for 10 min. Total cell lysates were immunoblotted with the indicated antibodies. (B) CytoQuant fluorescence assay for proliferation of mESCs treated with DMSO or DCA (20 µM or 100 µM). The data shown are means±SEM, n = 4). (C) Reversible effect of DCA on mESCs proliferation. After pretreatment with 100 µM DCA for 36 hrs, mESCs were washed with PBS 3 times and then cultured in the same medium but with DMSO, up to 72 hrs (Means±SEM, n = 4). (2.35 MB TIF) [file pone.0004914.s005.tif]
