# Supplementary material for: A Conserved Mechanism for Control of Human and Mouse Embryonic Stem Cell Pluripotency and Differentiation by Shp2 Tyrosine Phosphatase
Source: PLoS One. 2009 Mar 17;4(3):e4914. doi: 10.1371/journal.pone.0004914 (PMC2655646; doi:10.1371/journal.pone.0004914)
Supplement: Table S1 — DNA sequences of primers used for qRT-PCR. (0.06 MB DOC) [file pone.0004914.s006.doc]

| **Supplementary Table 1** | | |  |  |  |
| --- | --- | --- | --- | --- | --- |
|  |  |  |  |  |  |
| SPECI | SYMBOL | NAME | ACCESSION | forward primer | reverse primer |
| mouse | *Oct3/4* | POU domain, class 5, transcription factor 1 | NM_013633 | gttggagaaggtggaaccaa | ctccttctgcagggctttc |
| human | *OCT3/4* | POU domain, class 5, transcription factor 1 | NM_002701 | caatttgccaagctcctga | cgtttggctgaataccttcc |
| mouse | *Nanog* | Nanog homeobox | NM_028016 | ttcttgcttacaagggtctgc | agaggaagggcgaggaga |
| human | *NANOG* | Nanog homeobox | NM_024865 | atgcctcacacggagactgt | agggctgtcctgaataagca |
| mouse | *Sox2* | SRY (sex determining region Y)-box 2 | NM_011443 | gagagcaagtactggcaagaccg | tatacatggattctcgccagcc |
| human | *SOX2* | SRY (sex determining region Y)-box 2 | NM_003106 | atgggttcggtggtcaagt | ggaggaagaggtaaccacagg |
| mouse | *Rex1* | zinc finger protein 42 | NM_174900 | ggaagaaatgctgaaggtggagac | agtccccatccccttcaata |
| human | *REX1* | zinc finger protein 42 | NM_174900 | cagaacagaagaggccttcac | tctgagtaagctgtcttcagcaa |
| mouse | *Nestin* | nestin (Nes) | NM_016701 | ctgcaggccactgaaaagtt | gaccctgcttctcctgctc |
| human | *NESTIN* | nestin (Nes) | NM_006617 | tgcgggctactgaaaagttc | tgtaggccctgtttctcctg |
| human | *PAX6* | paired box gene 6 | NM_000280 | tcaccatggcaaataacctg | cagcatgcaggagtatgagg |
| mouse | *Desmin* | desmin | NM_010043 | gcgtgacaacctgatagacg | gttggatttcctcctgtagtttg |
| human | *DESMIN* | desmin | NM_001927 | ggagattgccacctaccg | ggtctggatggggagattg |
| mouse | *T* | brachyury (T) | NM_009309 | cagcccacctactggctcta | gagcctggggtgatggta |
| human | *T* | brachyury (T) | NM_003181 | acagcgcatgatcaccag | tttgcaaatggattgtacttaatttt |
| mouse | *Gata4* | GATA binding protein 4 | NM_008092 | actatgggcacagcagctc | ggacagcttcagagcagaca |
| human | *GATA4* | GATA binding protein 4 | NM_002052 | ggaagcccaagaacctgaat | ctggagttgctggaagcac |
| mouse | *Mef2c* | myocyte enhancer factor 2C | NM_025282 | ggtgctgacgggaacaac | cagttttcaatgcttttgttgg |
| mouse | *Myf5* | myogenic factor 5 | NM_008656 | cgccgtagcaggctgtgagttggcg | ctggctcttcaggacacagcttc |
| human | *SOX17* | SRY-box containing gene 17 | NM_022454 | acgccgagttgagcaaga | tctgcctcctccacgaag |
| human | *SHP2* | protein tyrosine phosphatase, non-receptor type 11 | NM_002834 | tggagattttgttctttctgtgc | ccaacgtcgtatttcagttcc |
| mouse | *Id1* | inhibitor of DNA binding 1 | NM_010495 | gcgagatcagtgccttgg | ctcctgaagggctggagtc |
| human | *ID2* | inhibitor of DNA binding 2 | NM_002166 | gcagcacctcatcgactaca | aattcagaagcctgcaagga |
| mouse | *Id3* | inhibitor of DNA binding 3 | NM_008321 | gaggagcttttgccactgac | gagagagggtcccagagtcc |
| human | *ID3* | inhibitor of DNA binding 3 | NM_002167 | ctggacgacatgaaccactg | gtagtcgatgacgcgctgta |
| mouse | *Cph* | peptidylprolyl isomerase A(cyclophilin A) | NM_008907 | caccgtgttcttcgacatc | attctgtgaaaggaggaacc |
| human | *CPH* | peptidylprolyl isomerase A(cyclophilin A) | [NM_021130](http://www.ncbi.nlm.nih.gov/entrez/viewer.fcgi?db=nucleotide&val=114520617) | ctcctttgagctgtttgcag | caccacatgcttgccatcc |
